# Dydrogesterone as an Option in the Medical Treatment of Endometriosis: A Brief Comment

**DOI:** 10.1055/s-0042-1751075

**Published:** 2022-07-15

**Authors:** Bruno Ramalho de Carvalho

**Affiliations:** 1 Bruno Ramalho – Reprodução Humana, Brasília, DF, Brazil

Dear Editor,

Endometriosis-related pain and morbidity are assumed to cause substantial impact on quality of life. Due to its chronicity, the disease is expected to be clinically managed in a long-term individualized plan, considering easy access, cost-effectiveness, acceptance, and adherence, regardless of surgical interventions, when (and if) they are necessary.

A better health-related quality of life is the main goal of the endometriosis treatment. It may be reached, individually, by relieving chronic pelvic pain, either acyclic, dysmenorrhea or dyspareunia, reducing the use of analgesics, and eventually protecting fertility. The optimized treatment of the disease often combines hormonal and surgical approaches, but the current literature 1 shows an inclination towards the use of medical strategies as first choices, ultimately postponing, or even avoiding, the need for surgery.

Aiming to inhibit the growth of endometriotic lesions, medical alternatives like selective progesterone receptor modulators, antiangiogenic factors and immunomodulators have been studied in recent years. 2 As a matter of fact, estrogens combined with progestagens or progestagens alone still seem to be the most used agents to suppress ovarian function and disease activity, 2 but there is a lack of comparative information to determine a best choice among the available molecules. Despite the different potencies to suppress the hypothalamic-pituitary-ovarian axis, progestagens in general may be similarly effective to control endometriosis. Individualization of the treatment is, then, expected to be the less expensive, with minimal adverse effects and maximal adherence. 3 4

Dydrogesterone is a retroprogesterone characterized by high selectivity for progesterone receptors and potent progestagenic activity. According to the medical leaflet, it may be prescribed in two regimens in the treatment of endometriosis, namely: a cyclical regimen, from the 5th to the 25th days of the menstrual cycle, and a continuous one, both with daily doses ranging from 10 mg to 30 mg. 5 However, knowledge on the comparative efficacy of the two protocols is also scarce, and the recently published ORCHIDEA Study 1 brought interesting data to light.

As the primary outcome, a significant decrease in the intensity of chronic pelvic pain was observed among women receiving dydrogesterone in the daily doses of 20 mg or 30 mg. By assessing women experience after treatment cycle 6, it was also observed a reduction in the number of days of analgesics use, and the severity of dysmenorrhea, as much as improvements in sexual well-being.1 In other words, the study pointed to a comparable gain in quality of life between the two daily doses, whether in cyclical or continuous regimens, at least for the first 6 months. 4

Regarding adverse events, the ORCHIDEA Study 1 found mild uterine bleeding as the most frequent one (1.1%), as it is expected for other progestagen-only treatment regimens, especially in the continuous model. However, despite being related to a better control of uterine bleeding, vaginal discharge and irritation, and coital well-being, than other progestagens, adverse events like headache, dizziness, abdominal pain, flatulence, and nausea may be more frequent with dydrogesterone, 6 and this must be better evaluated in future well-designed studies.

The earlier results of the application of dydrogesterone in women with endometriosis were published more than four decades ago, 7 but such a use has been notedly explored with more interest in the last fifteen years. The study by Trivedi et al. (2007) 8 was the first to convincingly demonstrate significant improvements in pelvic pain, dysmenorrhea and dyspareunia, and a 74% rate of satisfaction in the postlaparoscopic follow-up at 3 to 6 months.

A recently published meta-analysis 9 of 19 studies (1,709 women) confronted different regimens of dydrogesterone to gonadotropin-releasing hormone (GnRH) agonists, aromatase inhibitors or anti-progestagens against endometriosis. Despite the suggestion that dydrogesterone is the most effective among them for dysmenorrhea, little could be obtained regarding definite conclusions, since the available studies are generally small, non-randomized and heterogeneous. Of note, a special view on the efficacy of dydrogesterone to treat sexual dysfunction in women with endometriosis is expected from large cohorts with long term follow ups, since the preliminary data are encouraging. 10 Following the same reasoning, the ability of dydrogesterone to prevent the increase in size of ovarian endometriomas must be reassured by robust studies. 11

It is true that the absence of clinically relevant activity on estrogen, glucocorticoid, mineralocorticoid, or androgenic receptors may be a positive characteristic of dydrogesterone in currently recommended daily doses versus other progestagens. In addition to favoring the lower occurrence of adverse events, that pharmacological profile may be safer for women in childbearing age, in the absence of contraception. 6 12 Moreover, the good oral bioavailability and the theoretical lower risk of developing breast or endometrial cancer compared to other progestagens 6 are aspects to be considered for a first clinical treatment choice.

However, it should be argued that any progestagen may be initially considered sufficient against the symptoms of endometriosis. Then, what is expected from science is to fill the gaps by the confrontation of different progestins commonly used for treating endometriosis, such as the contemporary dienogest, 13 or the molecules from previous generations.

The information available to date is still insufficient to establish the clinical superiority of one molecule over the others. The ORCHIDEA Study 1 reported that dydrogesterone may relieve endometriosis-related chronic pelvic pain for at least 6 months of use in the regimen of preference. As aforementioned, the best progestagen for the treatment of endometriosis will be the one that is affordable, efficient, and well-accepted by each woman. Therefore, dydrogesterone is as welcome as any other progestagen in the search for the best individualized approach.
